# Effects of Cotton Seed Powder as the Seed Medium Nitrogen Source on the Morphology and Pneumocandin B_0_ Yield of *Glarea lozoyensis*

**DOI:** 10.3389/fmicb.2018.02352

**Published:** 2018-10-10

**Authors:** Ping Song, Kai Yuan, Xiao-Jun Ji, Lu-Jing Ren, Sen Zhang, Jian-Ping Wen, He Huang

**Affiliations:** ^1^School of Chemical Engineering and Technology, Department of Biochemical Engineering, Tianjin University, Tianjin, China; ^2^College of Biotechnology and Pharmaceutical Engineering, Nanjing Tech University, Nanjing, China; ^3^Jiangsu Collaboration Innovation Center of Chinese Medical Resources Industrialization, College of Pharmacy, Nanjing University of Chinese Medicine, Nanjing, China; ^4^School of Pharmaceutical Sciences, Nanjing Tech University, Nanjing, China

**Keywords:** pneumocandin B_0_, nitrogen source, morphology, enzyme activity, dissolved oxygen

## Abstract

Pneumocandin B_0_ is an important antifungal drug precursor produced by filamentous fungus *Glarea lozoyensis*. The high broth viscosity of cultures of this organism results in lower oxygen solubility and higher energy consumption for agitation and aeration, which mostly caused by the morphologies of filamentous fungi in submerged culture. In this study, the effects of different seed medium nitrogen sources on morphology and fermentation behavior of *G. lozoyensis* were investigated, and cotton seed powder resulted in small, compact pellets. Moreover, pneumocandin B_0_ yield in Erlenmeyer flasks were increased by 22.9%. Furthermore, pneumocandin B_0_ yield in a 50-L fermenter reached 2,100 mg/L and the dissolved oxygen maintained above 30%. Additionally, activities of phosphofructokinase (PFK), isocitrate dehydrogenase (ICDH), glucose 6-phosphate dehydrogenase (G6PDH), and malic enzyme (ME) were increased by 87.5, 50, 41.6, and 10.7%, respectively. This study demonstrates the feasibility and advantages of using cotton seed powder for controlling the fungal morphology and improving the product yield in pneumocandin fermentations.

## Introduction

Pneumocandin B_0_ is a cell-associated lipopeptide produced by the filamentous fungus *G. lozoyensis*, which is used as an intermediate in the synthesis of caspofungin acetate (Balkovec et al., 2013). Due to the high failure rates during management of fungal infections and increasing resistance problems in clinical practice caused by the overuse of polyenes (amphotericin B), azoles, and flucytosine, caspofungin became popular as soon as it was approved for human therapy (Denning, 2003). However, it is difficult to scale up the production of pneumocandin B_0_ to industrial levels due to high medium viscosity and pseudoplastic flow behavior in stirred-tank bioreactors caused by the morphology of *G. lozoyensis*. Then cultures become oxygen-limited and result in low product yield (Pollard et al., 2002). In order to meet the demand of 20% dissolved oxygen (DO) in pneumocandin B_0_ production, the energy consumption for agitation and aeration is increased during its industrial fermentation (Pollard et al., 2007). In addition to reducing energy consumption, DO can be increased by controlling the morphology of filamentous fungus.

Morphological engineering, defined as “tailoring morphologies for specific bioprocesses” (Mcintyre et al., 2001), encompasses all actions aimed at controlling fungal morphology (Antecka et al., 2016). Morphological engineering techniques include varying the spore concentration, pH-shifting, mechanical stress exerted by stirring and aeration, and regulating medium osmolarity (Nielsen et al., 1995; Papagianni, 2004; Cheng et al., 2009; Wucherpfennig et al., 2011). Furthermore, the utilized nitrogen source directly influences the hyphal morphology (Cho et al., 2002; Tanoi et al., 2011). In the case of the culture with feather-like morphology obtained using soybean meal, the arachidonic acid yield was two times higher than in culture grown using yeast extract, with circular pellet morphology (Park et al., 1999). Similarly, when soybean meal hydrolysate was used as the nitrogen source for *Rhizopus oryzae* fermentations, uniformly dispersed mycelial clumps with a diameter of 0.1 mm were formed, giving high fumaric acid production (Zhang et al., 2015). In addition, controlling the morphology by adding microparticles to the culture broth is gaining attention. For example, the addition of tale or aluminum oxide microparticles, *Aspergillus terreus* (Gonciarz and Bizukojc, 2014), *Rhizopus oryzae* (Coban and Demirci, 2016) or *Trichoderma atroviride* (Etschmann et al., 2015) formed small pellets, with high production yield. Therefore, different morphological forms of filamentous fungi, such as free mycelia, “hairy” pellets or loose mycelial clumps, have a vital influence on different specific products (Ahamed and Vermette, 2009; Krull et al., 2013). The advantages and disadvantages of mycelial vs. pellet form cultivation should therefore be carefully balanced for each biological system.

In this study, nitrogenous substances with or without microparticles were applied in pneumocandin B_0_ seed culture with the aim to find the most suitable nitrogen source to control morphology. The relationship of nitrogen source, PMV, morphology and pneumocandin B_0_ yield was investigated. Furthermore, in order to explore whether the pneumocandin B_0_ biosynthesis mechanism changed along with the change of morphology, we investigated the activities of four key enzymes involved in three main metabolic pathways. This study demonstrates the feasibility and advantages of using cotton seed powder as a low-cost nitrogen source for controlling the morphology and improving the product yield in pneumocandin fermentations.

## Materials and Methods

### Strain and Culture Medium

*Glarea lozoyensis* (CCTCC M 2014416) was preserved in the China Center for Type Culture Collection.

The initial seed medium consisted of (per liter): glucose 40 g, soybean powder (Beijing Hongrunbaoshun Technology Co., Ltd., China) 20 g, KH_2_PO_4_ 1 g, trace element solution 10 ml; the initial pH was adjusted to 5.0. The trace element solution was described in our previous study (Qin et al., 2016).

Other nitrogen sources include cotton seed powder (Beijing Hongrunbaoshun Technology Co., Ltd., China), cotton seed protein (Beijing Hongrunbaoshun Technology Co., Ltd., China), yeast extract (Thermo Fisher Scientific Inc., United States), tryptone (Thermo Fisher Scientific Inc., United States), soy peptone (Beijing Aaoboxing Bio.Tech Co., Ltd., China) and corn meal (Shandong Runyin Biological Chemical Co., Ltd., China) and are filtered by 80 mesh sifter.

The initial fermentation medium consisted of (per liter): glucose 20.0 g, D-mannitol 100 g, soy peptone (Beijing Aaoboxing Bio.Tech Co., Ltd., China) 20 g, K_2_HPO_4_ 2.5 g; the initial pH was 6.8.

### Culture Conditions

In case of fermentation in Erlenmeyer flasks, the cells growing on Potato Dextrose Agar Medium (PDA) slants were transferred to 250-mL Erlenmeyer flasks containing 50 ml seed medium and cultivated at 25°C on a shaker at 220 rpm for 5 days. Then, 10% (v/v) of the preculture was used to inoculate 50 ml of fermentation medium, which was cultured at 25°C on a shaker at 220 rpm for 17 days.

In case of fermentations in the fermenter, the preculture was cultivated in a 10-L seed flask (Shanghai Guoqiang Biochemical Engineering Equipment Co., Ltd., China) containing 5 L seed medium at 25°C. 120 h later, 10% (v/v) of the preculture was used to inoculate a 50-L production fermenter (Shanghai Guoqiang Biochemical Engineering Equipment Co., Ltd., China) containing 30 L of culture broth and cultivated at 25°C for 17 days. The agitation and aeration rates were set at 250 rpm and 1 vvm, respectively.

### Analytical Methods

Packed Mycelial Volume (PMV) was determined based on 5 ml cell suspensions harvested by centrifugation at 8,000 ×*g* for 10 min. Biomass was determined from 5 ml cell suspensions harvested by centrifugation, washed with distilled water, and dried at 80°C until constant weight (48 h).

The analytical methods used to determine the concentrations of D-mannitol and pneumocandin were described in our previous work (Qin et al., 2016). D-mannitol was analyzed using a Shimadzu HPLC system (Shimadzu LC-20AB, Shimadzu CTO-20A, and Shimadzu RID-10A, Shimadzu Corporation, Japan), using an Aminex HPX-87P column (300 mm × 7.8 mm, Bio-Rad, United States). Pneumocandin B_0_ was analyzed using a Dionex HPLC system (Dionex P680 pump, Chromeleon controller, and Dionex UVD 170U Detector; Dionex Corporation, Sunnyvale, CA, United States) equipped with an ODS column (Venusil MP C18, 4.6 mm × 250 mm, Agela Corporation, Tianjin, China). The detection wavelength was 210 nm.

### Statistical Analysis

The data of the batch fermentation were presented as the averages of three parallel samples, and the error bars indicate the standard deviation (SD) from the means of triplicates (Qin et al., 2016).

## Results and Discussion

### The Effect of Different Seed Medium Nitrogen Sources on PMV and Pneumocandin B_0_ Yield

Preculture, fermentation and downstream process were considered as the important stages of complex bioprocess. Whereas the main studies were more concentrated on fermentation and downstream process, ignoring preculture, which may also lead to failure of the industrial scale-up course because the seed are not at the “optimum” age and physiological state (Zou et al., 2011). In practice, a range of criteria are used to assess the quality of seed. These include measurements relating to the amount of biomass as well as the physiological state determined by residual nutrient concentration, metabolic activity or morphological form (Cunha et al., 2002; Zou et al., 2011).

**FIGURE 1:**
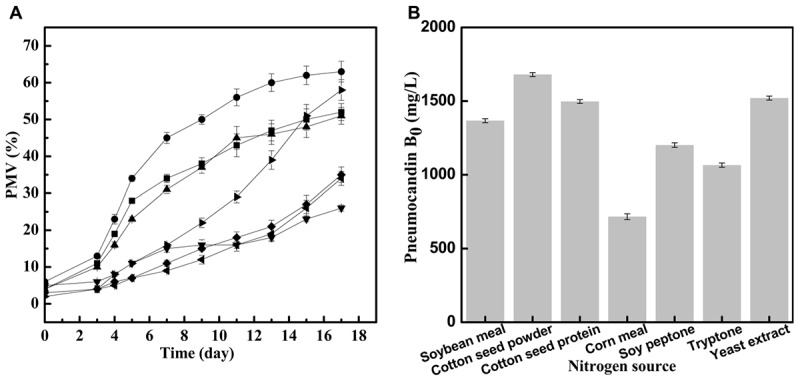
Different effects of nitrogen source on seed growth and pneumocandin B_0_ yield. **(A)** The PMV of *G. lozoyensis* grown in medium comprising different nitrogen sources: soybean meal 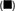, cotton seed powder 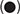, cotton seed protein 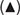, corn meal 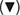, soy peptone 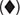, tryptone 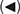, yeast extract 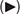. **(B)** The pneumocandin B_0_ yields of cultures harvested after 17 days of growth by preculture with different nitrogen sources.

The effect of different seed medium nitrogen sources on the growth of *G. lozoyensis* was evaluated to understand the relationship with cell growth during cultivation and to increase biomass productivity, hence improving the economics of pneumocandin B_0_ production. The changes of the growth rate of *G. lozoyensis* in seed medium with different nitrogen sources and PMV during 17 days of incubation are illustrated in **Figure 1A**. During the first period (days 0–3), there were no obvious differences of PMV with 7 different nitrogen sources. After 3 days, with the synthesis of key enzymes and the utilization of the corresponding substrates activated, the cells started to grow and display different growth rates.

With cotton seed powder as seed medium nitrogen source, the PMV exhibited a rapid growth between day 3 and day 13, and finally reached 63% at 17 days, which was better than the results obtained with any of the other tested nitrogen sources. Interestingly, when cotton seed protein was used as seed medium nitrogen source, the final PMV was 51%, implying that the effect of cotton seed protein was inferior to that of cotton seed powder. When yeast extract was used as seed medium nitrogen source, the strains grew slowly between day 0 and day 9 but grew rapidly during days 9–17, with the PMV reaching 58% in the end. Similar to cotton seed protein, the PMV in the soybean meal group increased quickly during days 3–7 and started to increase slowly during days 7–17, with a final PMV of 52%. By contrast, when tryptone, soy peptone and corn meal were used as seed medium nitrogen sources, the final PMV reached 35, 34, and 26%, respectively. These complex nitrogen sources, as with cotton seed powder or yeast extract, contain different amino acids, vitamins and other growth stimulating compounds (Tuliakova et al., 2004), but it is obvious that they are not optimal for the growth of *G. lozoyensis*.

Next, we investigated the effects of the seven different preculture on pneumocandin B_0_ yield in **Figure 1B**. The maximum pneumocandin B_0_ yield reached 1,680 mg/L with cotton seed powder as seed medium nitrogen source, and the lowest pneumocandin B_0_ yield was 700 mg/L when corn meal was used as seed medium nitrogen source. The highest pneumocandin B_0_ yield and PMV proved that as an intracellular metabolite, high PMV is advantageous for the accumulation of pneumocandin B_0_. The pneumocandin B_0_ yield with yeast extract as seed medium nitrogen source, one of the typical nitrogen sources for the culture of microorganisms, was 1,520 mg/L, and the PMV was 58%, only second to cotton seed powder. By contrast, many other complex nitrogen sources, including soybean meal, corn meal, soy peptone and tryptone, inhibited cell growth, resulting in lower pneumocandin B_0_ yield.

Even though yeast extract exhibited a similar effect on cell growth and pneumocandin B_0_ biosynthesis as cotton seed powder, its price was 62.5% higher than that of the latter. Therefore, among the various complex nitrogen sources, low-cost cotton seed powder was found to be the most favorable for the economical cultivation of *G. lozoyensis*.

### Effects of Different Seed Medium Nitrogen Sources on Hyphal Morphology

**FIGURE 2:**
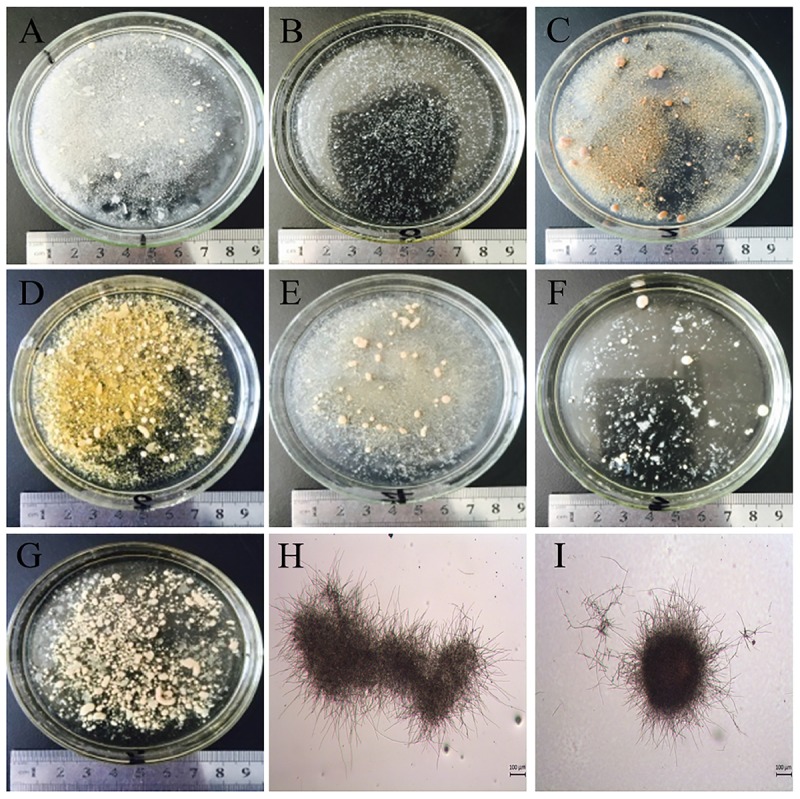
The macroscopic and microscopic morphology of *G. lozoyensis* after cultivation for 11 days in medium with different nitrogen sources with or without microparticles. Pictures **(A–G)** were taken using a normal camera and **(H,I)** were observed using a light microscope (LEICA DM 1000, Germany). **(A,H)** Soybean meal (loose mycelia and clumps), **(B,I)** cotton seed powder (small, uniformly compact pellets), **(C)** cotton seed protein (non-uniformly loose pellets), **(D)** corn meal (large compact pellets), **(E)** soy peptone (non-uniformly compact pellets), **(F)** tryptone (dispersed mycelia and pellets), **(G)** yeast extract (rod-shaped mycelial aggregates).

The microscopic observation of *G. lozoyensis* grown in the different medium revealed the presence of freely dispersed mycelia, mycelial clumps, or aggregates and pellets (**Figure 2**), illustrating that the nitrogen sources influenced the morphogenesis of the fungus. When soybean meal was used as nitrogen source without microparticles, *G. lozoyensis* formed loose mycelia and clumps (**Figures 2A,H**). By contrast, non-uniformly compact pellets were formed in the group grown on soy peptone without microparticles (**Figure 2E**). In the medium containing cotton seed protein without microparticles, non-uniformly loose pellets were formed (**Figure 2C**). When tryptone without microparticles was used as nitrogen source, *G. lozoyensis* was more likely to form dispersed mycelia and pellets (**Figure 2F**). With yeast extract without microparticles, rod-shaped mycelial aggregates were formed (**Figure 2G**). Subsequently, we analyzed the relationship between the morphology and pneumocandin B_0_ yield.

One of main morphologies was loose clumps or pellets with dispersed mycelia. These morphologies were more likely to increase the broth viscosity, resulting in difficulties with mixing, as well as limitations of the supply of oxygen and nutrients for the biomass (Metz, 1976). These adverse factors inhibited *G. lozoyensis* growth and reduced the pneumocandin B_0_ yield (**Figure 1B**).

The other main morphology was compact pellets with different sizes, which has been reported as a desired morphology for the production of itaconic acid (Metz, 1976), citric acid (Steel et al., 1954), or penicillin (König et al., 2010). Small, uniformly compact pellets (diameter of 0.1–0.3 mm) (**Figures 2B,I**) were obtained with cotton seed powder as seed medium nitrogen source in conjunction with microparticles, while large compact pellets (diameter of 1–2 mm) (**Figure 2D**) were formed when corn meal was used as seed medium nitrogen source in conjunction with microparticles. In the culture comprising pellets with a diameter of 1–2 mm, the pneumocandin B_0_ production was 700 mg/L, which was only approximately 41.7% of the maximum yield (1,680 mg/L). This result was in good agreement with another study (Du et al., 2003) on the morphology change of *Rhizopus chinensis* 12. The highest antibiotic production was accompanied by small pellets and large pellets resulted in a low antibiotic productivity. As a strict aerobe, *G. lozoyensis* requires sufficient DO and a high oxygen uptake rate during fermentation. Cultures with the compact pellet morphology had the lowest suspension viscosity and showed a broth rheology close to Newtonian flow behavior, with more DO. With the low solubility of oxygen, about 8.69 mg/L in water at 25°C, only small pellets can be fully penetrated by oxygen and enable a higher mass transfer (Driouch et al., 2012). When pellets have a diameter > 0.5 mm, an anaerobic core forms inside the pellet, limiting the mass and oxygen transfer (Roa Engel et al., 2011; Zhou et al., 2011). In *G. lozoyensis* fermentations with small pellets, the low suspension viscosity diminishes the non-oxygenated zone of the broth and the small pellets contribute to better availability of oxygen by increasing effective diffusivity. And this hypothesis would be worth examination in latter fermentation.

In conclusion, the results indicated that morphology, greatly affected by the nitrogen source, had a direct effect on cell growth and fermentation productivity. When cotton seed powder was applied as nitrogen source in the cultivation medium, small and compact pellets formed, and the obstacles, such as high broth viscosity and limited oxygen transfer were significantly reduced or even eliminated. This is one reason why the cells grew rapidly and had a high productivity in the culture with cotton seed powder.

### Optimization of Cotton Seed Powder Concentration and Preculture Cultivation Time on Pneumocandin B_0_ Yield

**FIGURE 3:**
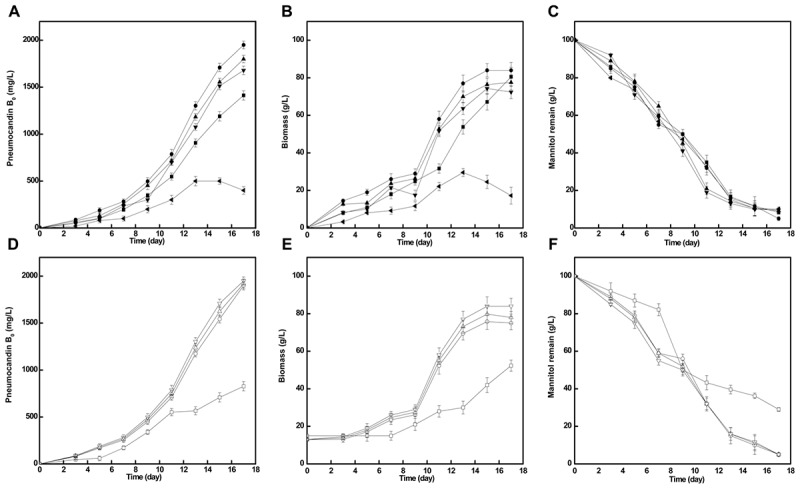
Effects of cotton seed powder concentration and preculture cultivation time on fermentation. The pneumocandin B_0_ **(A)**, biomass **(B)**, and mannitol remain **(C)** with different cotton seed powder concentration, 5 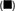, 10 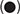, 15 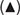, 20 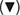, and 30 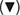 g/L. The pneumocandin B_0_ **(D)**, biomass **(E)**, and mannitol remain **(F)** with different inoculation time. The preculture were inoculated at 2nd (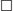), 3rd (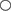), 4th (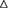), and 5th (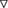) days.

The addition of different concentration of cotton seed powder to seed medium has an obvious influence on pneumocandin B_0_ yield. As shown in **Figure 3**, when cotton seed powder concentration was 10 g/L, pneumocandin B_0_ production reached a maximum yield (1,950 mg/L). With other cotton seed culture concentration (5, 15, 20 g/L), the pneumocandin B_0_ yield and biomass were lower. Especially when the cotton seed powder concentration was 30 g/L, the yield and biomass were only 400 mg/L and 17.23 g/L, which may caused by the insolubility of cotton seed powder, resulting in the weaken oxygen transfer.

At the same time, we further investigated the impact of inoculation time on pneumocandin B_0_ yield. During this part, we took inoculation after seed was cultivated 2nd, 3rd, 4th, and 5th day. As shown in **Figure 3**, except the inoculation after 2 days cultivation, the other three parallel groups were nearly similar in pneumocandin B_0_ yield (1,900, 1,920, 1,950 mg/L). Therefore, preculture was take inoculation after 3 days cultivation.

### Fermentation Results Using the Optimized Seed Nitrogen Source in a Scale-up Fermenter

**FIGURE 4:**
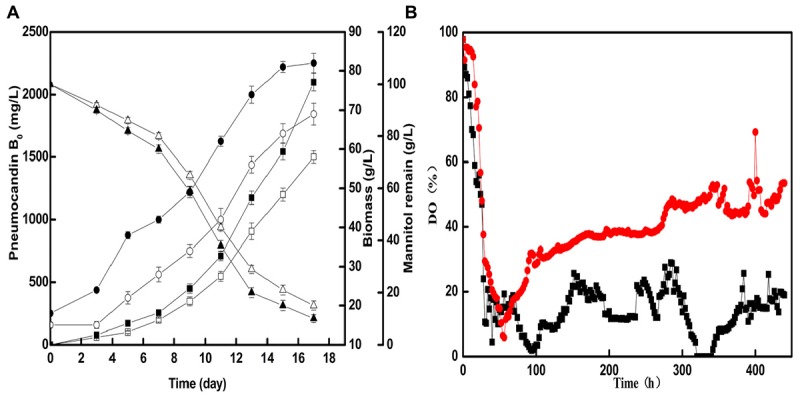
The fermentation situations of control group and optimized group in 50-L fermenter: **(A)** The effects of cotton seed powder as seed medium nitrogen source on the pneumocandin B_0_ yield (control group: 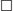, optimized group: 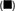, biomass (control group: 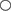, optimized group: 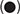, and remaining mannitol (control group: 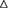, optimized group: 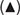. **(B)** Trends of DO (%) in two 50-L fermenter (control group: 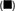, optimized group: 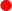).

Based on the optimal seed cultivation and fermentation, a fed-batch fermentation was successfully established in a 50-L fermenter to explore the effect of optimized preculture on the industrial pneumocandin B_0_ yield. The control group used soy peptone as the seed medium nitrogen source and the optimized group used cotton seed powder as the seed medium nitrogen source. As shown in **Figure 4A**, the fermentation showed a noticeable improvement with cotton seed powder as seed nitrogen source. The biomass and mannitol consumption were obviously increased during fermentation. At the end of fermentation, the biomass obtained with cotton seed powder was 82 g/L, corresponding to an 18.8% increase over the control group (69 g/L). At the same time, the remaining mannitol concentration of the optimized group was only 10 g/L, while in the control group it was 15 g/L. The maximal pneumocandin B_0_ yield in the optimized group was 2,100 mg/L, which was increased by 40% with respect to the control group (1,680 mg/L). A series studies on improving pneumocandin B_0_ yield were carried out in recent years. Li et al. (2014) and Qin et al. (2016) both optimized the medium and the pneumocandin B_0_ yield were reached 1,138 and 1,873 mg/L, respectively. Their works focused on the effects of different medium distribution ratios on pneumocandin B_0_ yield. While this study explores the reason that why the nitrogen source, cotton seed powder, has positive effect on pneumocandin B_0_ yield. Finally, the result indicates that morphology of *G. lozoyensis* plays a vital role in pneumocandin B_0_ fermentation.

Next, we investigated the changes of DO (%) during fermentation. As shown in **Figure 4B**, after the optimized of preculture morphology, with the same agitation and aeration, the DO of optimized group was higher and more stable than control group. During the phase of former fermentation, *G. lozoyensis* grown and mannitol was essentially used for the synthesis of catalytic biomass, with high oxygen consumption. Therefore, the DO (%) rapidly decreased from 97.8 to 10.2 and from 97.8 to 5.9 in control and optimized group, respectively. During the phase of latter fermentation, morphology of *G. lozoyensis* in control group was formed loose mycelia and clumps, may resulted high broth viscosity and oxygen uneven distribution. But the morphology of *G. lozoyensis* in optimized group was formed small, compact pellets, with low broth viscosity, well oxygen dissolved and transfer. At the late stage of logarithmic phase, although the biomass of optimized group was approximately 32% higher than the control group, the DO of the optimized group could be maintained above 30%, while the DO of the control group was below 20% and not stable. It shows that different morphologies have a great influence on DO. This result is agreed with a study on *Aspergillus niger* (Hille et al., 2005). The morphology of bulk or pellet influenced the DO directly and pellet size influenced oxygen transfer. This proved the hypothesis that the morphology of small, compact pellets is suitable for aerobic *G. lozoyensis* fermentation and is advantages to oxygen dissolved and transfer. And this may be part reason of high pneumocandin B_0_ yield.

**FIGURE 5:**
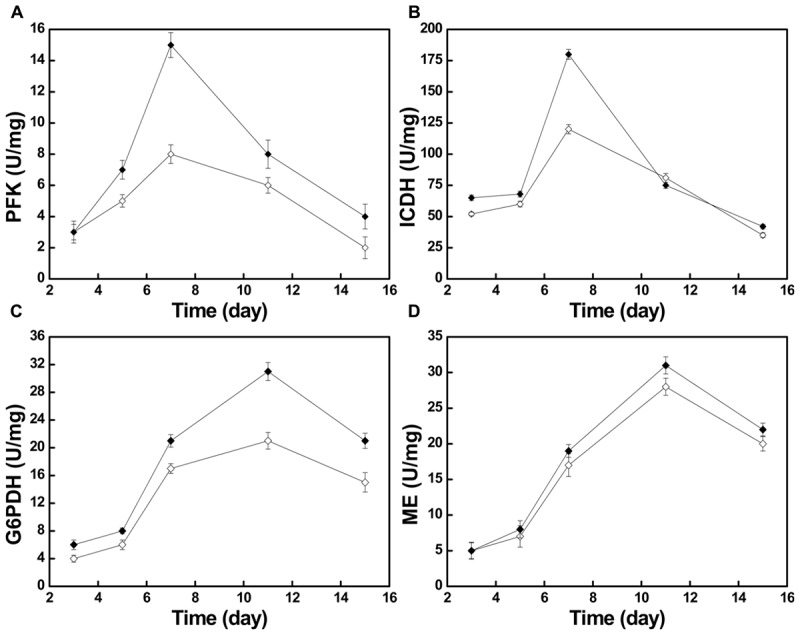
The changes of different key enzyme activities in the control group (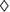) and the optimized group 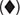. PFK activity **(A)**, ICDH activity **(B)**, G6PDH activity **(C)**, and ME activity **(D)**.

Moreover, we investigated some of the changes of metabolic pathway activity by measuring the activities of four key enzymes, phosphofructokinase (PFK), isocitrate dehydrogenase (ICDH), glucose 6-phosphate dehydrogenase (G6PDH), and malic enzyme (ME). As shown in **Figure 5**, after the nitrogen source was changed, the activities of the four key enzymes were all higher than in the control group during the entire fermentation, the only exception being the activity of ICDH, one of the key enzymes of tricarboxylic acid cycle (TCA cycle), at day 11.

The activities of PFK and ICDH, which can be used as proxies for the activity of the Embden–Meyerhof–Parnas pathway (EMP) and the TCA cycle, respectively, both kept increasing during days 3–7 (**Figures 5A,B**). Especially on day 7, during the exponential phase, the PFK activity reached its maximum of 15 U/mg in the optimized group and 8 U/mg in the control group. During the same time, the ICDH activity reached its maximum of 180 U/mg in the optimized group and 120 U/mg in the control group. The increase of PFK and ICDH activity indicates that the increased activity of the EMP and TCA cycle offered more energy and building blocks for growth (**Figure 1**). During days 3–7, both strains showed a vigorous increase of biomass.

With the gradual depletion of the nitrogen source, the activity of adenosine monophosphate deaminase (AMPD) will increase, which results in phosphate transfer from adenosine monophosphate (AMP) to inosine monophosphate (IMP) (Evans and Ratledge, 1985). ICDH is AMP-dependent, and its activity may be reduced or it can be inactivated at decreasing AMP concentrations (Botham and Ratledge, 1979). During days 7–11, with consumption of the nitrogen source, ICDH activity declined rapidly in the optimized group (**Figure 5B**). This led to a stagnation of the TCA cycle and the massive accumulation of isocitrate in the mitochondria. The accumulated isocitrate is converted to citric acid and transported through the malate/citrate transporter on the mitochondrial membrane to the outside of the mitochondria (Palmieri et al., 1996). Then, under the action of ATP:citrate lyase, it is cleaved to form acetyl-CoA, the key two-carbon metabolite in several metabolic processes, particularly in terms of precursor supply for the 10R,12S-dimethylmyristylside chain of pneumocandin B_0_, which determines the start of peptide elongation, directly affecting pneumocandin B_0_ biosynthesis (Chen et al., 2016). In addition to the sufficient supply of acetyl-CoA, it is well known that the supply of reducing power in form of NADPH has a critical effect on fatty acid biosynthesis (Sun et al., 2016). Earlier work (Adefarati et al., 1991; Palmieri et al., 1996) indicated that NADPH plays a significant role in the biosynthesis of pneumocandin B_0_, whereby ME may work synergistically with G6PDH to provide reduced NADPH in filamentous fungi (Hong et al., 2013; Li et al., 2013; Hao et al., 2014).

During days 3–11, the activities of ME and G6PDH kept increasing and both reached their maxima at day 11 (**Figures 5C,D**). The activity of G6PDH in the optimized group reached 31 U/mg at day 11, which compared favorably to the 21 U/mg of the control group, representing a 47.6% increase. Similarly, the activity of ME at day 11 was 31 U/mg in the optimized group and 28 U/mg in the control group. During days 5–11, the biosynthesis rate of pneumocandin B_0_ started to increase, which may have been caused by the high supply of NADPH from ME and G6PDH. At the same time, the mannitol concentration continued to decrease quickly, implying that the strains were in an active growth phase.

Taken together, after change of *G. lozoyensis* morphology, the strain’s growth and pneumocandin B_0_ biosynthesis were improved by influencing the broth viscosity, oxygen transfer, and key metabolic pathways. These observations explain why the final yield of pneumocandin B_0_ reached 2,100 mg/L in the 50-L fermenter, demonstrating that cotton seed powder is a highly valuable seed medium nitrogen source for *G. lozoyensis* fermentations.

## Conclusion

In this study, the effects of different nitrogen sources on *G. lozoyensis* morphology and pneumocandin B_0_ yield were investigated. With cotton seed powder, a beneficial morphology with small, uniformly compact pellets was formed, which resulted in a 40% increase of pneumocandin B_0_ production in a 50-L fermenter. At the same time, the DO can maintain above 30%. Moreover, the enzyme activities of PFK, ICDH, G6PDH, and ME were increased by 87.5, 50, 41.6, and 10.7%, respectively. In conclusion, cotton seed powder can be used as a low-cost nitrogen source for improving pneumocandin B_0_ production in industrial fermentations employing *G. lozoyensis*.

## Author Contributions

PS and KY carried out the experiments and wrote the manuscript. X-JJ provided technical support of scale-up fermentation. L-JR revised the manuscript. SZ provided detection of pneumocandin B_0_. J-PW and HH designed this work and agreed to be accountable for all aspects of the work.

## Conflict of Interest Statement

The authors declare that the research was conducted in the absence of any commercial or financial relationships that could be construed as a potential conflict of interest.
